# Comparative proteomic analysis of extracellular vesicles isolated from porcine adipose tissue-derived mesenchymal stem/stromal cells

**DOI:** 10.1038/srep36120

**Published:** 2016-10-27

**Authors:** Alfonso Eirin, Xiang-Yang Zhu, Amrutesh S. Puranik, John R. Woollard, Hui Tang, Surendra Dasari, Amir Lerman, Andre J. van Wijnen, Lilach O. Lerman

**Affiliations:** 1 Divisions of Nephrology and Hypertension, Mayo Clinic, Rochester, MN, USA; 2 Health Sciences Research, Mayo Clinic, Rochester, MN, USA; 3 Cardiovascular Diseases, Mayo Clinic, Rochester, MN, USA; 4 Orthopedic Surgery, Mayo Clinic, Rochester, MN, USA

## Abstract

Extracellular vesicles (EVs) isolated from mesenchymal stem/stromal cells (MSCs) contribute to recovery of damaged tissue. We have previously shown that porcine MSC-derived EVs transport mRNA and miRNA capable of modulating cellular pathways in recipient cells. To identify candidate factors that contribute to the therapeutic effects of porcine MSC-derived EVs, we characterized their protein cargo using proteomics. Porcine MSCs were cultured from abdominal fat, and EVs characterized for expression of typical MSC and EV markers. LC-MS/MS proteomic analysis was performed and proteins classified. Functional pathway analysis was performed and five candidate proteins were validated by western blot. Proteomics analysis identified 5,469 distinct proteins in MSCs and 4,937 in EVs. The average protein expression was higher in MSCs vs. EVs. Differential expression analysis revealed 128 proteins that are selectively enriched in EVs versus MSCs, whereas 563 proteins were excluded from EVs. Proteins enriched in EVs are linked to a broad range of biological functions, including angiogenesis, blood coagulation, apoptosis, extracellular matrix remodeling, and regulation of inflammation. Excluded are mostly nuclear proteins, like proteins involved in nucleotide binding and RNA splicing. EVs have a selectively-enriched protein cargo with a specific biological signature that MSCs may employ for intercellular communication to facilitate tissue repair.

Mesenchymal stem/stromal cells (MSCs) are principal components of an important endogenous repair system that allows constant self-renewal of adult tissues in many organs. MSCs were first described in 1970 as colony forming units fibroblasts^1^ and can be isolated from various tissues, including bone marrow, adipose tissue, umbilical cord blood, and peripheral blood. Although MSC populations have been characterized by different sets of markers, specific consensus criteria have been defined to identify MSCs, including adherence to plastic in standard culture conditions, expression of common MSC cell surface markers (e.g. CD73, CD90, and CD105), and capacity for trilineage differentiation into osteoblasts, adipocytes, and chondrocytes in cell culture^2^. In addition to their ability to migrate and differentiate into multiple cell linages, MSCs exert unique pro-angiogenic, anti-inflammatory, and immuno-modulatory effects that promote tissue repair and regeneration. These characteristics of MSCs are attractive for their application in cell-based therapies for tissue regeneration.

Over the last decade, MSCs have been employed to ameliorate tissue injury and accelerate repair in a wide variety of disorders. One particularly attractive application is in cardiovascular disease, the leading cause of mortality in countries with developed and emerging economies^3^. Indeed, a progressively increasing number of studies has focused on the ability of MSCs to repair the damaged myocardium in murine models of hypertension and heart failure^4^,^5^. Furthermore, in recent years, we and others have tested the efficacy of MSCs for attenuating cardiac and renal injury in large animal models of cardiovascular disease^6^. For example, intra-myocardial delivery of MSCs safely reduces infarct size and improves cardiac function in porcine ischaemic heart failure^7^. Furthermore, we have shown that intra-renal adjunctive MSC-based therapy with or without restoration of renal vascular patency improves renal and cardiac function in porcine renovascular disease, confirming the safety and efficacy of this intervention^8^,^9^,^10^,^11^.

Accumulating data indicate that MSCs release large amounts of extracellular vesicles (EVs) that mediate their paracrine effects by shuttling different types of RNAs (e.g., mRNA and miRNA) and proteins to recipient cells^12^,^13^. Consistent with this concept, we recently found that porcine MSC-derived EVs contain a selective combination of mRNAs and miRNAs capable of regulating transcription of genetic information and modulating angiogenesis, adipogenesis, and other pathways in recipient cells^14^. To support the potential utility of EVs in augmenting MSC-related therapies, the protein expression profile of porcine MSC-derived EVs needs to be characterized. In this study, we elucidated the protein content of porcine MSC-derived EVs to gain important insight into the molecular mechanisms by which they exert paracrine actions and contribute to the reparative potency of MSCs. Using a quantitative proteomic strategy to study the protein cargo of porcine MSC-derived EVs, we identified candidate proteins and biological signatures that are consistent with the postulated therapeutic effects of MSCs.

## Materials and Methods

### MSC and EV characterization and culture

All experiments were performed in accordance with the guidelines and approval of the Mayo Clinic Institutional Animal Care and Use Committee. MSCs were isolated from subcutaneous abdominal fat (5–10 g) of 3 female domestic pigs, as previously described^14^. Cells were cultured for 3 weeks in advanced MEM medium (Gibco/Invitrogen) supplemented with 5% platelet lysate (PLTmax, Mill Creek Life Sciences, Rochester, MN) in 37°/5% CO_2_, and the third passage collected for *in vitro* phenotypic and functional analyses. MSCs expressed CD44, CD90, and CD105 markers, and differentiated into osteocytes, chondrocytes, and adipocytes, as previously described^8^,^9^,^15^, consistent with our experience with human MSCs^16^.

EVs were obtained from supernatants of MSCs using the ultra-centrifugation method^14^. In brief, 10 × 10^6^ MSCs (a dose used for *in vivo* injections)^8^,^9^,^10^,^11^ were cultured for 48 h in advanced MEM medium without supplements and centrifuged at 2,000 g. Cell-free supernatants were then subjected to ultra-centrifugation at 100,000 g for 1 h at 4 °C, washed in serum-free medium containing HEPES 25 mM, and submitted to a second step of ultracentrifugation.

Transmission electron microscopy (TEM) of MSC supernatant with 2% uranyl acetate negative staining was performed, and cup-shaped 40–1000 nm structures identified as EVs. Micrographs were taken on a digital electron microscopy (JEOL 1200 EXII). EVs were further characterized based on the expression of EV (CD9, CD29, and CD63) and MSC (CD105 and CD73) surface markers by western blotting. In addition, EV size distribution was assessed by nanoparticle tracking analysis (NTA) using NanoSight NS300. EVs were diluted with PBS and samples continuously run through a flow-cell top-plate at 25 μL/min. Three videos (120 seconds each) of Brownian motion of nanoparticles were recorded and 1,000 completed tracks analyzed using NTA 2.3.5.

### Proteomic profiling and network pathway analysis

Liquid chromatography mass spectrometry (LC-MS/MS) proteomic analysis was performed at the Mayo Clinic Proteomics Core, as previously described^17^,^18^. All MSC and EV pellets were solubilized in Tris buffer with 1%SDS HALT protease inhibitor and Benzonase. Lysis was achieved with by rapid agitation (‘vortexing’) followed by three consecutive 5 min incubations on ice. Protein samples were then denatured by incubation at 85 °C for 10 min. Aliquots with a protein equivalent of 20 μg were dried down and resolubilized in reducing sample buffer. Samples were electrophoresed in 4–20% TGX Ready gels at 200 V for 30 min.

The gel lanes were divided into 6 evenly spaced horizontal regions using as guides protein bands that were common to all samples. Each sample lane was cut length-wise in the middle to obtain technical duplicates. The resulting individual gel sections were diced and transferred into PCR tubes for digestion and extraction. Gel sections were digested with trypsin following a previously described protocol^18^. In brief, each gel section was de-stained and the proteins present in the section were reduced with dithiothreitol and alkylated with iodoacetamide. Proteins were digested by overnight incubation at 37 °C with 140 ng of trypsin dissolved in 25 mM Tris (pH 8.2). Peptides were extracted from the gel piece with 30 μL of 50% acetonitrile in 4% trifluoroacetic acid, followed by two additional extractions with 50 μL aliquots of acetonitrile. The combined extracts were evaporated to dryness on a vacuum concentrator and stored at −80 °C until further analysis.

Peptide extracts from each gel section were reconstituted in 40 μL HPLC-grade water containing 0.2% formic acid, 0.1% trifluoroacetic acid, and 0.002% Zwittergent 3–16. Aliquots of the peptide extracts (typically 10 μL, but 15 μL for the two gel sections encompasses higher molecular weight proteins) were loaded onto a 0.25 μL bed OptiPak trap (Optimize Technologies, Oregon City, Oregon) custom-packed with 5 μm, 200 Å Magic C8 (Bruker-Michrom, Auburn, CA) in the stationary phase. The loaded trap was washed for 4 min with an aqueous loading buffer of 0.2% formic acid and 0.05% trifluoroacetic acid at a flow rate of 10 μL/min. Following the wash, peptides were transferred via a 10-port valve onto a 35 cm × 100 μm PicoFrit column 9 (NewObjective, Woburn, MA), self-packed with Agilent Poroshell 120S 2.7 μm EC-C18 stationary phase, using a Dionex UltiMate 3000 RSLC LC system (Thermo-Fisher Scientific, Waltham, MA). Peptides were separated using a 400 nL/min LC gradient comprised of 2–30%B in 0–70 min, 30–50%B from 70–100 min, 50–95%B from 100–104 min, held at 95%B for 8 min and re-equilibrated to 2%B. Mobile phase A was 2% acetonitrile in water with 0.2% formic acid and mobile phase B was acetonitrile /isopropanol/water (80/10/10 by volume) with 0.2% formic acid. Eluting peptides were analyzed using a QExactive mass spectrometer (Thermo-Fisher Scientific, Waltham, MA). The instrument was operated in data-dependent mode by collecting MS1 data at 70,000 resolving power (measured at m/z 200) with an AGC value of 3E6 over an m/z range of 350–2000, using lock masses from background polysiloxanes at m/z 371.10123 and 445.12002. Precursors were fragmented with normalized collision energy of 27, and fragments measured at 17,500 resolving power and a fixed first mass of 140. Tandem mass spectra (MS/MS) were collected for the top 15 precursor masses present in each MS1 using an AGC value of 1E5, maximal ion fill time of 100 ms, an isolation window of 3.0 Da, isolation offset of 0.5 Da, and a dynamic exclusion time of 60 s.

We utilized a label-free peptide MS1 intensity-based method for finding differentially expressed proteins between experimental groups. The quality of the raw data was assessed using the quality control metrics in the Swift proteomic data processing pipeline. MaxQuant (version 1.5.1) software processed the raw data files to produce a list of protein groups and their corresponding intensities in each sample^19^. To accomplish this, MaxQuant was configured to use a composite porcine protein sequence database containing UniProt porcine reference proteome (downloaded on 08/12/2015) and sequences of common contaminants (trypsin, keratin, cotton, wool, etc.). Reversed protein sequences were appended to the database for estimating protein identification false discovery rates (FDRs). The software was configured to use 20 ppm m/z tolerance for precursors and fragments while performing peptide-spectrum matching. The software derived semi-tryptic peptides from the sequence database while looking for the following variable modifications: carbamidomethylation of cysteine (+57.023 Da.), oxidation of methionine (+15.994 Da.), formation of n-terminal pyroglutamic acid (−17.023 Da.) and protein n-terminal acetylation (+42.01 Da.). MaxQuant was instructed to align the runs and match features between multiple sample runs of the same gel region. The software filtered peptide and protein identifications at 1% FDR, assembled protein identifications into groups and reported protein group intensities.

An in-house R-programming script performed differential expression analysis using protein group intensities. First, protein group intensities of each sample were log_2_ transformed and normalized using a trimmed mean (5%) method. For each protein group, the normalized intensities observed in two groups of samples were modeled using a Gaussian-linked generalized linear model. ANOVA was used to detect the differentially expressed protein groups between pairs of experimental groups. Differential expression analysis was performed after the data was normalized by protein loading, and differential p-values FDR-corrected using the Benjamini-Hochberg-Yekutieli procedure^20^. Protein groups with an FDR < 0.05 and an absolute fold change (EVs/MSCs) > 10 were classified as enriched in EVs, whereas those with FDR < 0.05 and fold change <−10 were considered as excluded.

Proteins enriched in or excluded from EVs were classified by their molecular function, cellular localization, and class, using Protein Analysis Through Evolutionary Relationships (PANTHER)^21^. Functional pathway analysis was performed using the Database for Annotation and Integrated Discovery (DAVID 6.7)^22^.

### Validation of proteomic analysis

Complement component (C2), vascular endothelial growth factor (VEGF), and von Willebrand factor (vWF) proteins, which were all enriched in EVs, were selected for validation, and their expression in EVs and MSCs measured by Western blot (Novus: Cat#NBP1-58985, Santa Cruz Biotech: cat# sc-152, and Abcam: cat#ab6994, respectively). Similarly, we validated the expression of RNA polymerase-associated protein RTF1 homolog (RTF1) and FOS-Like Antigen 1 (FOSL1) excluded from EVs (Abcam: cat#ab99362 and LifeSpan BioSiences, Inc. cat#:aa130-179, respectively).

## Results

**Figure 1:**
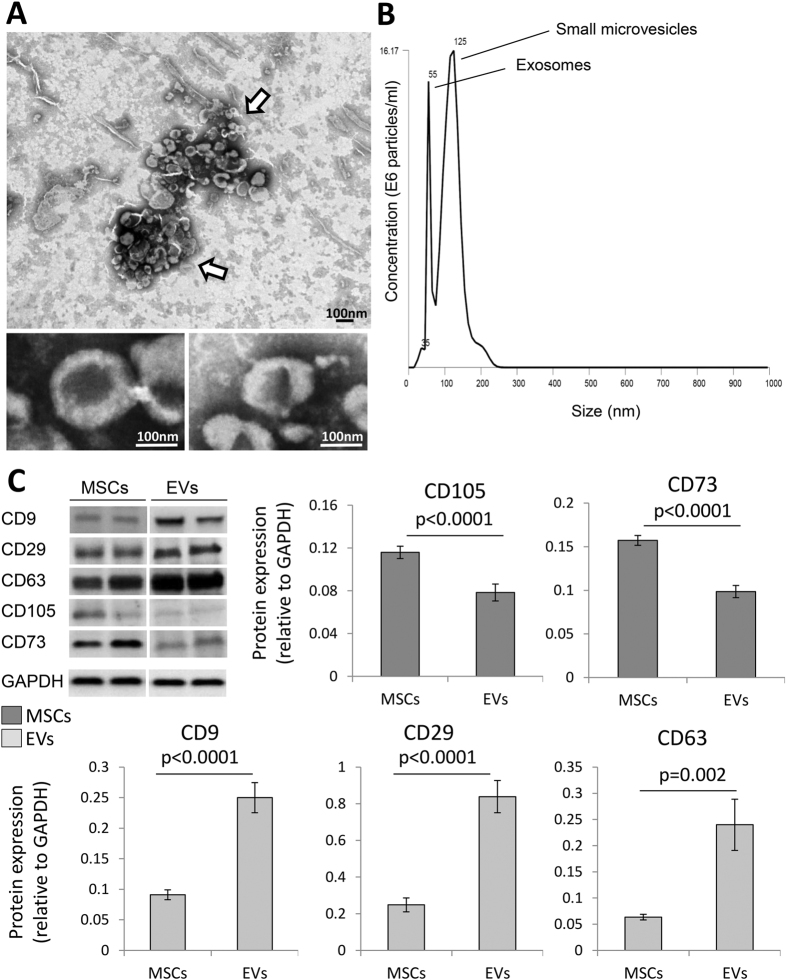
(**A**) Transmission electron microscopy of MSC culture supernatants (negative staining) showing EV clusters (arrows) with the classic “cup-like” morphology. (**B**) Size distribution of isolated EVs revealed a composition of about 2/3 small microvesicles (~125 nm in size) and 1/3 exosomes (~55 nm). (**C**) MSC and EV protein expression of common MSC (CD105 and CD73) and EV (CD9, CD29, and CD63) markers.

Cultured MSCs released significant amounts of EVs with a classic “cup-like” morphology (Fig. 1A), most of which were under 300 nm in size with a bi-phasic size-distribution (Fig. 1B) consistent with a composition of approximately 2/3 small microvesicles (~125 nm) and 1/3 exosomes (~55 nm). The EV expressed primarily EV (CD9, CD29, and CD63) and MSC surface markers (CD105 and CD73) (Fig. 1C).

### Identification and classification of differentially expressed proteins

**Figure 2:**
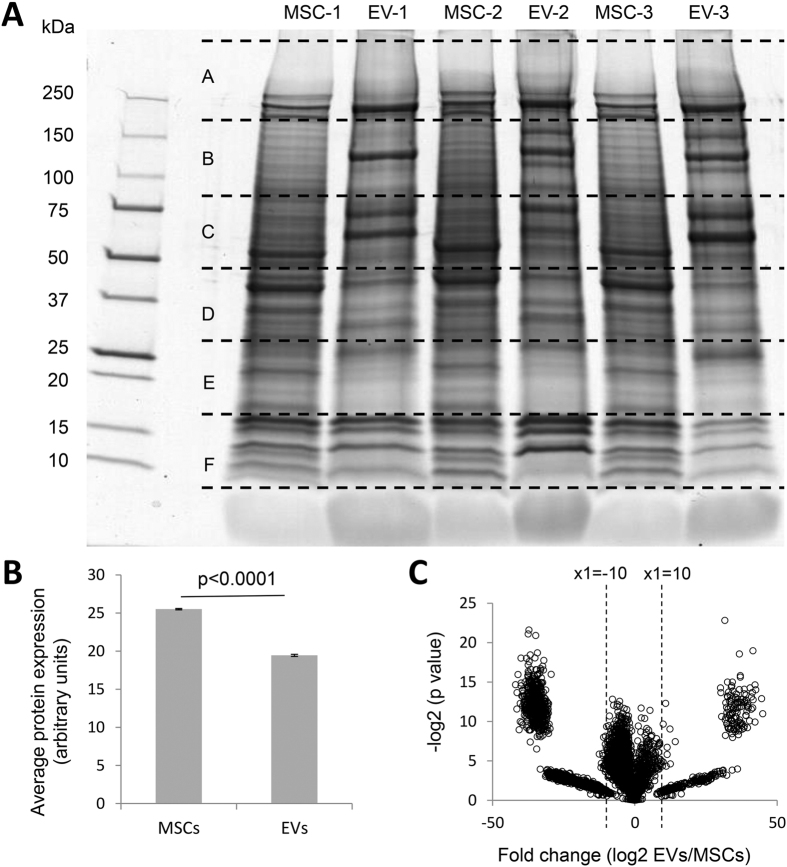
(**A**) Master gel for LC-MS/MS proteomic analysis of 3 MSC and 3 EV samples. The gel shows the relative molecular weight on the left side. Slices were labeled (**A**–**F**) with A being at the highest molecular mass >250 kDa and F being the lowest (10–15 kDa). (**B**) Average protein expression was higher in MSCs compared to EVs. (**C**) Volcano plot of identified proteins in MSCs and EVs.

Proteomics analysis identified peptides derived from a total of 5,469 distinct protein groups in MSCs and 4,937 in EVs, with molecular weights ranging from 10–250 kDa (Fig. 2A). Average protein expression was higher in MSCs compared to EVs (Fig. 2B, p < 0.0001). EVs expressed 12 MSC and 77 EV markers (Tables 1 and 2). Differential expression analysis revealed 128 proteins upregulated in EVs vs. MSCs (Table S1, fold change EVs/MSCs >10, p < 0.05), whereas 573 proteins were excluded from EVs (fold change EVs/MSCs < −10, p < 0.05) (Fig. 2C).

### Proteins enriched in EVs

**Figure 3:**
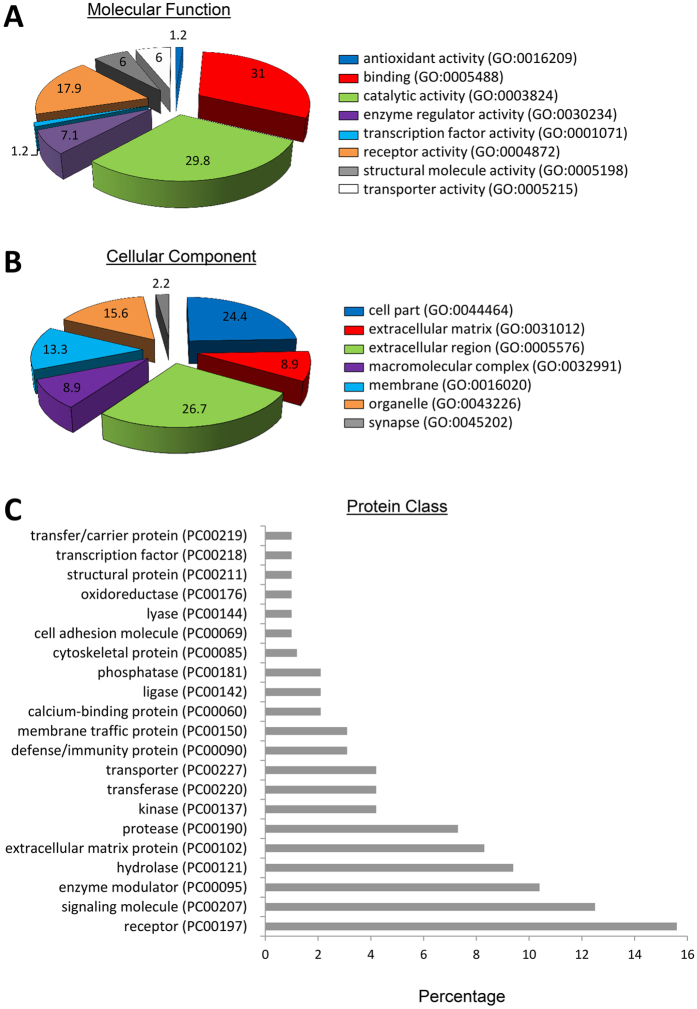
Panther analysis of molecular function (**A**) cellular component (**B**) and class (**C**) of proteins upregulated in EVs compared to their parent MSCs.

**Figure 4:**
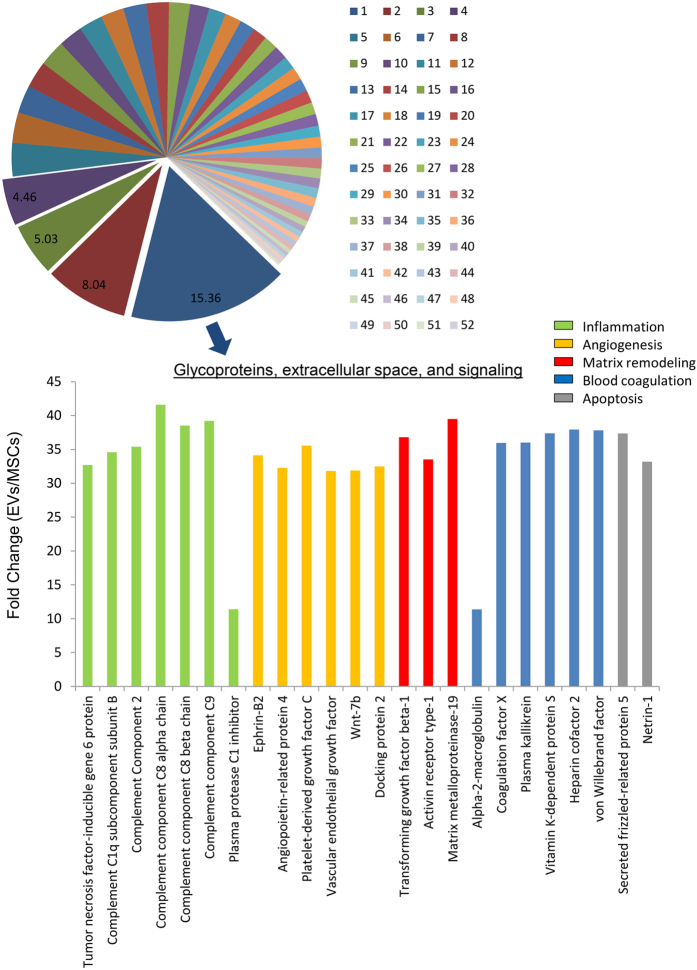
Functional annotation clustering (using DAVID 6.7) of proteins enriched in EVs (top) and histograms showing distribution by fold change of EV proteins in the Glycoproteins, extracellular space, and signaling category (bottom). 1: Glycoproteins, extracellular space and signaling; 2: Wound healing; 3: Extracellular matrix; 4: Adaptive immune response; 5: Heparin binding; 6: Protein interaction; 7: Regulation of inflammatory response; 8: Peptidase activity; 9: Vesicle proteins; 10: Vesicle-mediated transport; 11: Response to steroid hormone stimulus; 12: Complement pathway; 13: Peptidase activity; 14: Cell adhesion; 15: Lipid binding; 16: Epidermal growth factor pathway; 17: Response to extracellular stimulus; 18: Lipoprotein; 19: Respiratory development; 20: Exocytosis; 21: TGF-beta signaling pathway; 22: Enzyme inhibitor activity; 23: Cell proliferation; 24: Cell surface; 25: Cell differentiation; 26: Vesicle-mediated transport; 27: Cell adhesion; 28: Protein complex assembly; 29: Positive regulation of transport; 30: Cytokine-cytokine receptor interaction; 31: Intracellular signaling; 32: Angiogenesis; 33: Response to carbohydrate stimulus; 34: Basement membrane; 35: Phospholipidic metabolic process; 36: Plasma membrane; 37: Regulation of cell size; 38: Protein transport; 39: Nucleotide binding; 40: Response to hypoxia; 41: Membrane proteins; 42: Regulation of kinase activity; 43: Intracellular signaling; 44: Cell migration; 45: ATP binding; 46: Calcium binding; 47: Lysosome; 48: Chemical homeostasis; 49: Microtubule; 50: Apoptosis; 51: Immunoglobulin; 52: Cytoskeleton.

The majority of the proteins enriched in EVs showed binding, catalytic, and receptor activity, and were equally distributed between intracellular and extracellular compartment categories (Fig. 3A,B). A number of different classes of proteins were enriched in EVs, with receptors, signaling molecules, and enzyme modulators representing the most frequently occurring protein categories types (Fig. 3C). Functional classification showed a remarkable diversity of biological roles. A total of 52 clusters were identified, among which glycoproteins, extracellular space, and signaling proteins comprised the most relevant category (enrichment score = 15.4). From a biological perspective, these proteins are functionally involved in angiogenesis, blood coagulation, extracellular matrix remodeling, inflammatory response, and apoptosis (Fig. 4). These main categories were followed by closely related categories that encompass proteins involved in wound healing (enrichment score = 8.04), extracellular matrix (5.03), and adaptive immune response (4.46). The remaining categories were approximately equally distributed (average enrichment score = 1.2 ± 0.8).

### Proteins excluded from EVs

**Figure 5:**
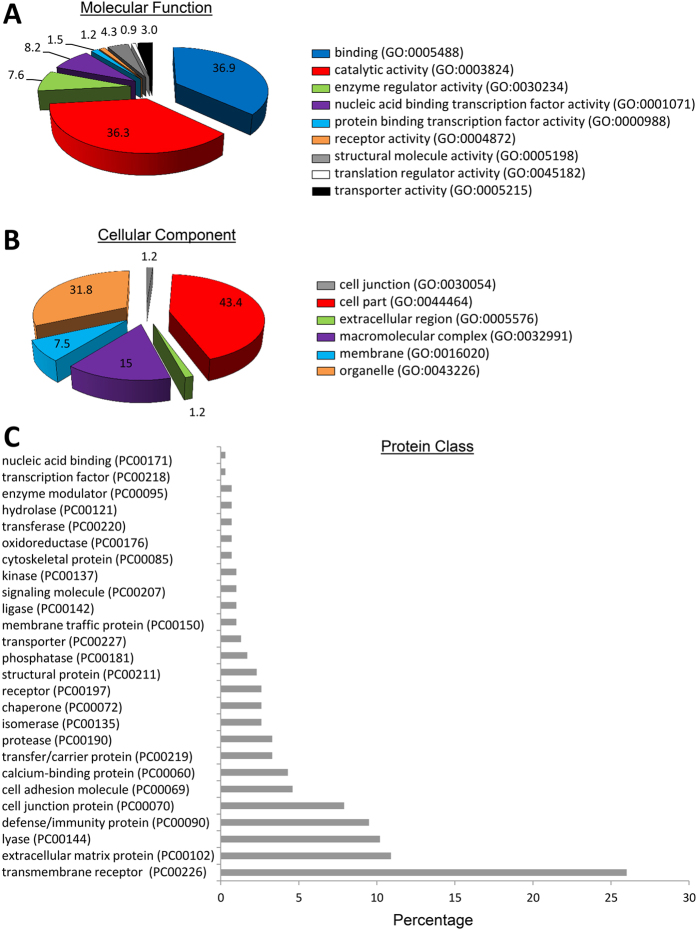
Panther analysis of molecular function (**A**) cellular component (**B**) and class (**C**) of proteins excluded from EVs.

**Figure 6:**
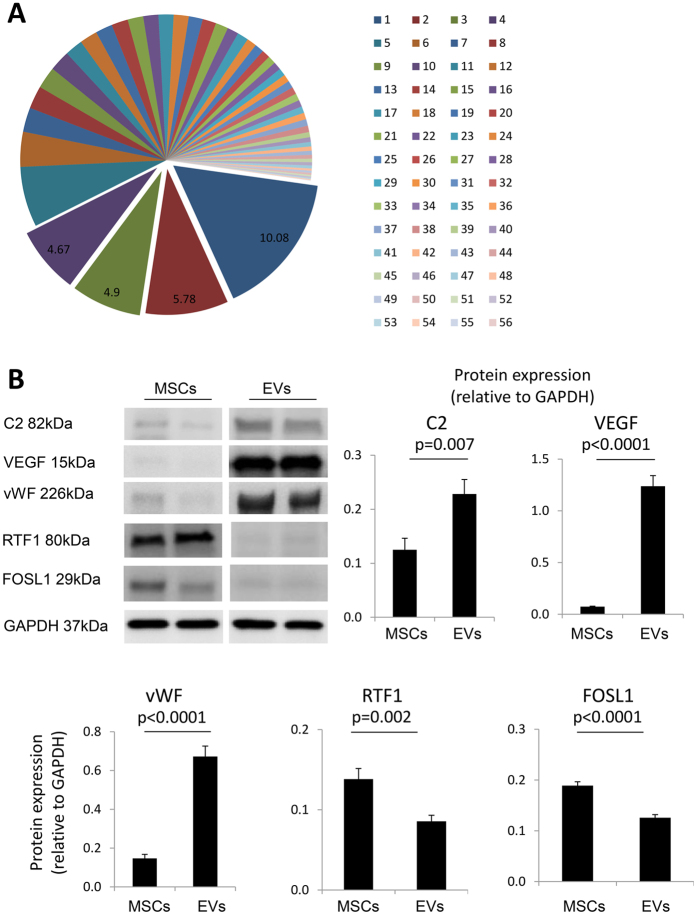
(**A**) Functional annotation clustering (using DAVID 6.7) of proteins excluded from EVs. 1: Nucleolus; 2: Nucleoplasm; 3: RNA splicing; 4: Nucleotide binding; 5: Nucleolus; 6: Histone modification; 7: DNA binding; 8: RNA binding; 9: Apoptosis; 10: FHA regulatory proteins; 11: Isopeptide bond; 12: N-acetyltransferase activity; 13: GTPase regulator activity; 14: ATP binding; 15: RNA transport; 16: DNA replication; 17: Mitochondria; 18: Ubiquitin protein activity; 19: Transcription factors; 20: Macromolecular complex assembly; 21: Lipid synthesis; 22: Vesicle-mediated transport; 23: Transcription cofactor activity; 24: Helicase activity; 25: Regulation of cell death; 26: Cell division; 27: Regulation of transferase activity; 28: DNA repair; 29: Respiratory development; 30: Eye development; 31: Mitochondrial membrane; 32: Protein transport; 33: Transcription regulator activity; 34: Signaling proteins; 35: Transcription repressor activity; 36: Zinc fingers; 37: Organelle membrane; 38: Protein kinase cascade; 39: Vasculature development; 40: Immune system development; 41: MAPkinase signaling pathway; 42: Endoplasmic reticulum; 43: WD40 repeat region; 44: Zinc finger; 45: GTP binding; 46: Magnesium ion binding; 47: Cytoskeleton; 48: Enzyme receptor signaling; 49: Calcium ion binding; 50: Spermatogenesis; 51: Response to hormone stimulus; 52: Cell migration; 53: Cytoplasmic vesicle; 54: Protein dimerization activity; 55: Peptidase activity; 56: Cell fraction. (**B**) Expression of the candidate proteins complement component (C2), vascular endothelial growth factor (VEGF), von Willebrand factor (vWF), RNA polymerase-associated protein RTF1 homolog (RTF1), and FOS-Like Antigen 1 (FOSL1) was in accordance to the proteomics findings.

Proteins excluded from EVs possessed binding and catalytic activity, and were mostly detected in key cell functions and organelles (Fig. 5A,B). The major functional class for these excluded proteins was the nucleic acid binding proteins, followed by transcription factors and enzyme modulators (Fig. 5C). Functional pathway analysis revealed their participation in a broad spectrum of cellular processes and subcellular locations (56 clusters). The most notable categories are nuclear proteins (enrichment score = 10.08), nucleoplasm (5.78), RNA splicing (4.9), nucleotide binding (4.67), and nucleolus (4.21) (Fig. 6A). The remaining 51 categories were equally distributed (average enrichment score = 0.7 ± 0.5).

### Validation of proteomic analysis

Expression of the candidate proteins followed the same patterns as the proteomics findings. Specifically, C2, VEGF, and vWF were higher in EVs compared to their parent MSCs, whereas RTF1 and FOSL1 were higher in MSCs (Fig. 6B).

## Discussion

In the current study, we describe the biological signature of porcine MSC-derived EVs from a proteomics perspective. Our analysis identified a large number of proteins enriched in EVs, bearing the potential to act as modulators of angiogenesis, blood coagulation, extracellular matrix remodeling, apoptosis, and inflammation. Moroever, we identified several nuclear proteins and proteins involved in nucleotide binding and RNA splicing that are selectively excluded from EVs. These observations provide important insights into the molecular mechanisms underpinning the reparative capacity of MSC-derived EVs and underscore the broad spectrum of factors present in EVs as mediators of MSC-related intercellular communication. Moreover, our findings might help develop novel regenerative therapies using MSC-derived EVs for tissue repair.

Accumulating evidence indicates that EVs are major effectors of the paracrine actions of MSCs, and mediate their cell-to-cell comunication. Bruno and colleagues demonstrated that EVs derived from human bone marrow MSCs stimulate proliferation *in vitro* and confer resistance to apoptosis in renal tubular epithelial cells by activating a proliferative program via horizontal transfer of mRNA^23^. MSC-derived EVs have also been shown to reduce infarct size in mice with myocardial ischemia/reperfusion injury^24^ and promote angiogenesis in rats with myocardial infarction^25^. These results clearly indicate that EVs contribute to the principal trophic effects of MSC transplantation.

We have recently characterized the mRNA and miRNA cargo of porcine MSC-derived EVs using high-throughput RNA sequencing analysis to elucidate nucleic acid-based mechanisms by which they exert their tissue protective properties^14^. We found that MSC-derived EVs contain a combination of mRNAs and miRNAs capable of regulating transcription of genetic information and modulating angiogenesis, adipogenesis, extracellular matrix turnover, TGF-β signaling, and other pathways in recipient cells. The current study extends our previous observations and demonstrates that MSC-derived EVs contain proteins capable of modulating several signaling pathways and gene networks, providing molecular evidence useful to elucidate the paracrine effects of MSCs. Of particular interest for the use of EVs in supporting cardiovascular repair, our current proteomic findings combined with our previous transcriptomic findings indicate that EVs are enriched for proteins, mRNAs and miRNAs that individually, and perhaps together, support angiogenesis.

LC-MS/MS analysis offers the possibility to quantitatively characterize multiple protein groups by combining the physical separation capabilities of LC with the mass analysis capabilities of MS. Our proteomic analysis show that EVs derived from porcine adipose tissue MSC express several prototypical MSC markers (e.g. CD44, CD73, and CD105), as well as 79 of the top 100 EV markers identified in the Exocarta database (http://www.exocarta.org/, e.g. CD29 and CD63). Notably, we identified 128 proteins enriched in EVs compared to MSCs. Funtional enrichment analysis of these proteins indicates high representation of glycoprotein, extracelular space, and signaling proteins involved in angiogeneis, inflammation, matrix remodeling, blood coagulation, and apoptosis.

Experimental studies have demonstrated the pro-angiogenic potential of MSCs both *in vivo* and *in vitro*, providing a mechanistic rationale for their therapeutic use in stimulating angiogenesis. We have previously shown that a single intra-renal delivery of MSC substantialy improved the porcine renal microvasculature, associated with increased renal expression of VEGF, a key angiogenic factor that promotes endothelial cell survival and vascular sprouting^8^. The current study shows that VEGF is enriched in EVs, associated with upregulation of Ephrin-B2, a transmembrane ligand for Eph receptor tyrosine kinases that promotes VEGF-induced angiogesis^26^. Therefore, EVs may not only deliver the VEGF protein, but also upregulate its production in recipient cells. Furthermore, the EV proteome also includes several other key proteins involved in angiogenesis, such as angiopoietin-related protein-4, platelet-derived growth factor-C (PDGFC), and Wingless-Type (wnt) MMTV Integration Site Family, Member 7B (WNT7B). Our observations are underscored by recent findings showing up-regulated angiogenic associated pathways in exosomes derived from human bone marrow MSCs^27^, illustrating the conservation of the nature of EV cargo across species. Indeed, using a Venn diagram analysis, we compared our findings with those reported by Anderson *et al*., and identified 1,362 common EV proteins, among which several are involved in vascular development and angiogenesis including angiopoietin-1, Wnt Family Member-5A, and Notch-1 (Figure S1). Overall, there incomplete (~70%) overlap of EV proteins between the studies may be due to differences in MSC origin, interspecies variability, culture medium, and EV isolation methods. Importantly, our study extends previous findings, as our LC-MS/MS analysis identified a larger number of EV proteins.

MSCs also possess potent immunosuppressive and anti-inflammatory properties, partly via non-specific anti-proliferative actions on a wide range of immune cells^28^. Interestingly, we found increased EV expression of several inflammatory mediators, particularly members of the complement system, which modulate adaptive immune responses by interacting with receptors on dendritic cells and lymphocytes^29^. Furthermore, the complement system mediates the migration of MSCs to sites of inflammation and tissue injury, enhancing its immuno-modulatory actions^30^.

Additionally, proteins involved in extracellular matrix remodeling were enriched in EVs compared to MSCs. These included matrix metalloproteinase 9 (MMP9), which is involved in the breakdown of extracellular matrix, but also secreted by MSC and downregulates the cytotoxicity of natural killer cells^31^,^32^. Thus, delivery of this enzyme in EVs may supplement MSC immuno-modulatory actions. Furthermore, TGF-β1 is enriched in EVs. We have previously shown that EVs contain high levels of several mRNAs that encode for protein ligands within the TGFβ family, including TGFB1, TGFB3, FURIN, and ENG^14^. TGFβ proteins are involved in tissue remodeling and fibrosis, but also induce CD4+/CD25+ regulatory T-cells, dampering the inflammatory response^33^. In addition, TGFβ induces release of cytochrome-c from mitochondria triggering apoptosis^34^. Interestingly, we observed upregulation in other proteins that modulate apoptosis, including netrin-1 and secreted frizzled-related protein (SFRP)-5^35^,^36^. These observations may therefore indicate a pro-apoptotic function for EVs, possibly to facilitate removal of damaged cells.

We also found robust EV expression of proteins involved in blood coagulation, such as vWF, coagulation factor X, and plasma kallikrein, which may reflect the innate procoagulant activity of MSCs. Consistent with this concept, a recent study showed that intracoronary MSC delivered during reperfusion in porcine may manifest pro-coagulant properties, emphasizing the need for caution when applying cell-based therapy^37^. Interestingly, our EVs express several members of the secretory component of platelets, including vWF, PDGFC, and TGFβ, possibly because MSCs were cultured in platelet lysate^38^. However, our MSCs do not express endothelial markers like CD31 and CD34^8^,^9^, arguing against contamination with endothelial cells. Further studies are needed to explore the mechanisms by which vWF is enriched in MSC-derived EVs.

Apart from enrichment, our results revealed selective exclusion of a number of proteins from EVs, such as nuclear proteins and proteins involved in nucleotide binding and RNA splicing, including nucleolar complex associated-4 homolog, cleavage and polyadenylation specific factor-3, and CWC15 spliceosome-associated protein homolog. The mechanisms that regulate packaging these molecules into EVs remain unclear, but evidently cells are definitely selective in determining what proteins emerge as cargo in EVs.

Our study is limited by the relatively low number of samples, given the labor and cost of obtaining EVs from porcine disease models and of proteomic analysis. Nevertheless, a similar number of samples is commonly used in comparable studies reporting cell/tissue proteomic profiles^39^,^40^. Furthermore, post-translational changes in MSCs and EVs proteins unrevealed by proteomic analysis were not be assessed here, although LC-MS/MS has high sensitivity and robust precision for detecting MSC and EV protein groups. Indeed, follow-up studies using western blots demonstrated the efficacy of our proteomic analysis to detect changes in representative protein expression between MSCs and the EVs they produce. Future studies are needed to confirm these findings and test whether the protein content of *in-vivo* MSCs correlates with our observations in cultured MSCs. In addition, additional studies may ascribe specific cargo in our EV to microvesicles versus exosomes.

In summary, having examined the proteome of porcine MSCs and EVs, this study identified a number of proteins that may contribute to the molecular mechanisms involved in MSC-mediated tissue repair. We identified 128 proteins enriched and 563 excluded from EVs. Importantly, proteins enriched in EVs can potentially participate in tissue repair and regeneration by modulating angiogenesis, blood coagulation, apoptosis, extracellular matrix remodeling, and inflammation, whereas those actively depleted from EVs were mostly nuclear proteins or involved in nucleotide binding and RNA splicing. Therefore, our findings provide the foundation for future studies that focus on tissue protective effects of MSC-derived EVs and may guide the development of novel EV-based therapies for several diseases.

## Additional Information

**How to cite this article**: Eirin, A. *et al*. Comparative proteomic analysis of extracellular vesicles isolated from porcine adipose tissue-derived mesenchymal stem/stromal cells. *Sci. Rep.* **6**, 36120; doi: 10.1038/srep36120 (2016).

**Publisher’s note:** Springer Nature remains neutral with regard to jurisdictional claims in published maps and institutional affiliations.

## Supplementary Material

Supplementary Information

## Figures and Tables

**Table 1:** MSC markers expressed in EVs.

Protein name	UniProt ID	Gene Name
5′-Nucleotidase/CD73	K7GSR6_PIG	NT5E
BMPR-II	K7GQF4_PIG	BMPR2
CD44	K7GM14_PIG	CD44
Endoglin/CD105	K7GQF2_PIG	ENG
Fibronectin	F1SS24_PIG	FN1
Integrin alpha 1/CD49a	F1SMF6_PIG	ITGA1
Integrin alpha 5/CD49e	F1SR53_PIG	ITGA5
Integrin alpha V/CD51	B5B2Z3_PIG	ITGAV
Integrin beta 1/CD29	F1RVE7_PIG	ITGB1
Nucleostemin	F1SH97_PIG	GNL3
TfR (Transferrin R)	D7RK08_PIG	TFRC
Vimentin	A0A0B8RVD8_PIG	VIM

**Table 2:** EV markers expressed in EVs.

UniProt ID	Gene Name	UniProt ID	Gene Name	UniProt ID	Gene Name
F1RRD6_PIG	PDCD6IP	I7HD36_PIG	ATP1A1	K9IVR9_PIG	KPNB1
G3P_PIG	GAPDH	F1SA98_PIG	YWHAQ	F1SB42_PIG	EZR
F1S073_PIG	ANXA2	FLOT1_PIG	FLOT1	F1SLC4_PIG	ANXA4
K7GST0_PIG	CD63	A0A0B8RSX6_PIG	FLNA	A0A0B8RW53_PIG	ACLY
F1RT83_PIG	SDCBP	A0A0B8S0A2_PIG	CLIC1	F2Z5N8_PIG	RAB14
A0A0B8RSY9_PIG	ENO1	CDC42_PIG	CDC42	A0A0B8RZJ8_PIG	GNB1
HS90A_PIG	HSP90AA1	D0G0C8_PIG	CCT2	A0A0B8RZ10_PIG	UBA1
A0A0B8S031_PIG	PKM	K9J6H8_PIG	A2M	F1SS26_PIG	THBS1
LDHA_PIG	LDHA	F2Z4Z1_PIG	YWHAG	F1RFQ7_PIG	RAN
F2Z558_PIG	YWHAZ	I3LFI0_PIG	RAC1	RAB5A_PIG	RAB5A
PGK1_PIG	PGK1	M3V7×9_PIG	LGALS3BP	F1SAY0_PIG	PTGFRN
I3LII3_PIG	EEF2	Q06AS6_PIG	GNAI2	I3LR32_PIG	CCT5
F2Z5C1_PIG	ANXA5	ANXA1_PIG	ANXA1	TCPG_PIG	CCT3
I3LC73_PIG	FASN	I3LVS7_PIG	RHOA	B0LY42_PIG	BSG
C0MHR2_PIG	CLTC	B2CZF8_PIG	MFGE8	SAHH_PIG	AHCY
Q007T3_PIG	CD81	F1SDX9_PIG	PRDX2	I3LFZ8_PIG	RAB5B
F1RUN2_PIG	ALB	F1RUK8_PIG	GDI2	I3LLG9_PIG	LAMP2
D0G7F6_PIG	TPI1	F1SSX0_PIG	EHD4	I3LEE5_PIG	ITGA6
F2Q9A3_PIG	PPIA	F1RI39_PIG	ACTN4	GELS_PIG	GSN
F1RTN3_PIG	MSN	F1SDR7_PIG	YWHAB	F1SS24_PIG	FN1
COF1_PIG	CFL1	F1SPG0_PIG	RAB7A	F2Z4Y1_PIG	YWHAH
F1S3U9_PIG	PRDX1	A5GFU2_PIG	GNAS	F1SR80_PIG	TUBA1A
F1RFY1_PIG	PFN1	D7RK08_PIG	TFRC	A8U4R4_PIG	TKT
F1RVE7_PIG	ITGB1	F2Z560_PIG	RAB5C	F1SB63_PIG	TCP1
F1RS36_PIG	HSPA5	M3VH45_PIG	ANXA6	F2Z5I8_PIG	RAB8A
I3LB80_PIG	SLC3A2	F1S2E2_PIG	ANXA11		
