# Supplementary material for: Comparative proteomic analysis of extracellular vesicles isolated from porcine adipose tissue-derived mesenchymal stem/stromal cells
Source: Sci Rep. 2016 Oct 27;6:36120. doi: 10.1038/srep36120 (PMC5081562; doi:10.1038/srep36120)
Supplement: Supplementary Information [file srep36120-s1.pdf]

Supplementary information

Comparative proteomic analysis of extracellular vesicles isolated from porcine adipose tissue-derived mesenchymal stem/stromal cells

Short title: Proteome of stem cell-derived vesicles

Alfonso Eirin[1], MD; Xiang-Yang Zhu[1], MD, PhD; Amrutesh S. Puranik[1], PhD; John R. Woollard[1], Hui Tang[1], MD, PhD; Surendra Dasari[2], PhD; Amir Lerman[3], MD; Andre J. van Wijnen[4], PhD; Lilach O. Lerman[1], [3], MD, PhD

Divisions of Nephrology and Hypertension[1], Health Sciences Research[2], Cardiovascular Diseases[3] and Orthopedic Surgery[4], Mayo Clinic, Rochester, MN, United States

Table S1. Proteins enriched in EVs (fold change EVs/MSCs >10, p<0.05)

UniProt ID	Gene Name	UniProt ID	Gene Name	UniProt ID	Gene Name
B0F9U1_PIG	HP	PGRP2_PIG	PGLYRP2	F1RLT6_PIG	GNAZ
F1STZ1_PIG	C1QB	A0A075B7I7_PIG		ARRC_PIG	ARR3
B0M1M8_PIG	PIGRAD54	F1SFW5_PIG	SLC27A3	I3LJC9_PIG	HAPLN3
I3LTB8_PIG		H9LBP0_PIG	SERPIND1	F1RU22_PIG	EFEMP2
F1RPQ9_PIG	LIPG	G5CZ05_PIG	VWF	B8XTR8_PIG	KLKB1
A0SEH1_PIG	C8A	F1RPT1_PIG	PSTPIP2	F1RN41_PIG	F10
K7GLQ6_PIG	GPIBB	I3LQM7_PIG	SLC38A5	I3LFH1_PIG	IGHA
SAMP_PIG	APCS	I3LAK9_PIG	ADAMTS2	F1RT53_PIG	LYN
F1S288_PIG	SNTB1	F1SMQ2_PIG	TSPAN33	GPX5_PIG	GPX6
K7GMZ8_PIG	CP	I3LQM5_PIG	PROS1	F1S8L1_PIG	JAKMIP1
F1SC68_PIG	ADAMTS4	F1S8X3_PIG	SFRP5	I3LL31_PIG	PDGFC
K7GT70_PIG	CD36	I3LCN1_PIG	ENO2	I3LJL8_PIG	RFTN1
F1SN31_PIG	SDPR	F1S7X2_PIG	MYCT1	F1SEJ8_PIG	VSTM4
A5GFX6_PIG	TUBB1	FCERG_PIG	FCER1G	F1SDH5_PIG	CSNK1G2
I3L8N7_PIG	LOC102166965	F1RQP7_PIG	RASGRP2	I3LMG3_PIG	BIN2
K7GT68_PIG	ITGA6	F1RW71_PIG	MMRN1	A0A0B8RW16_PIG	C2
K7GQ82_PIG	BTK	I3LDK6_PIG	TGFB1	Q75R37_PIG	SHAS2
A0SEH3_PIG	LOC100037955	I3LUX3_PIG	SLC7A11	F1S1C9_PIG	OLFML2B
F1SPJ1_PIG	MMP19	I3LG86_PIG	RNF13	Q9GKJ6_PIG	BCHE
I3LGN5_PIG		I3LBB6_PIG	PTPRJ	F1S3W5_PIG	MOB3C
A0SEG9_PIG	C9	F1SUL2_PIG	LRRC32	I3LBQ1_PIG	MVB12B
F1SFA1_PIG	PON1	Q1KS52_PIG	ALS	F1RUE4_PIG	GPLD1
F1RPA3_PIG	GPRC5B	A9Z0A7_PIG	FCN1	F1RQM7_PIG	PRSS35
F1RV50_PIG	TTYH2	Q9TV82_PIG	LMP7	F1S474_PIG	CMIP
A0A075B7I2_PIG		Q7YS66_PIG	CLAUDIN5	F1SQR3_PIG	NTN4
A0SEH2_PIG	C8B	I3LLX4_PIG	PLEKHB2	ICA_PIG	ICA
F1RK01_PIG	CPB2	F1SLP7_PIG	LOC100739671	ANGL4_PIG	ANGPTL4
I3LVM5_PIG	SKAP2	F1STR4_PIG	PRSS23	F1RRX4_PIG	LCP2

F1SRC9_PIG	CDCP1	K9IW76_PIG	ACVR1	I3LMQ6_PIG	NAALADL2
F1S8V7_PIG	CPN1	I3LKE0_PIG	LOC100525 936	I3LVH1_PIG	WNT7B
M3V816_PIG	SLC39A6	F1SFF0_PIG	PTGER2	Q8SPZ9_PIG	VEGFA
F1SVA2_PIG	TINAGL1	F1SUI0_PIG	NTN1	F1SI16_PIG	BMPR2
F1SPI7_PIG	TRH	F1S1M1_PIG	SYTL4	I3LIF4_PIG	UBTD2
Q28937_PIG		F1SNU5_PIG	LOC100516 109	F1RIA4_PIG	TMEM132A
B0LDS8_PIG	EFNB2	Q06AT7_PIG	RHOF	F1SMG2_PIG	FGF10
I3LJJ6_PIG		F1S006_PIG	FN3K	K7ZJP7_PIG	IGHM
F1S2H8_PIG	IQGAP2	I3LU23_PIG	ARL13B	I3L746_PIG	PLEK
I3LSP0_PIG	LOC102157777	F1RW19_PIG	DGKK	F1RQ01_PIG	FERMT3
I3LER9_PIG		I3L7U2_PIG	TNFAIP6	K7GMV3_PIG	SERPING1
I3LDR2_PIG	TUBA8	F1RI88_PIG	STX3	K7GMU4_PIG	A2M
B6ECP2_PIG	GPIBA	F1RVY9_PIG	UNC13D	B2CZF8_PIG	MFGE8
F1RUR3_PIG	CCNY	F1RMC2_PIG	DOK2	Q7JJ74_PIG	PTGFRN
F1RKE8_PIG	TESC	I3LDX0_PIG	SLC15A4		

Fig.S1

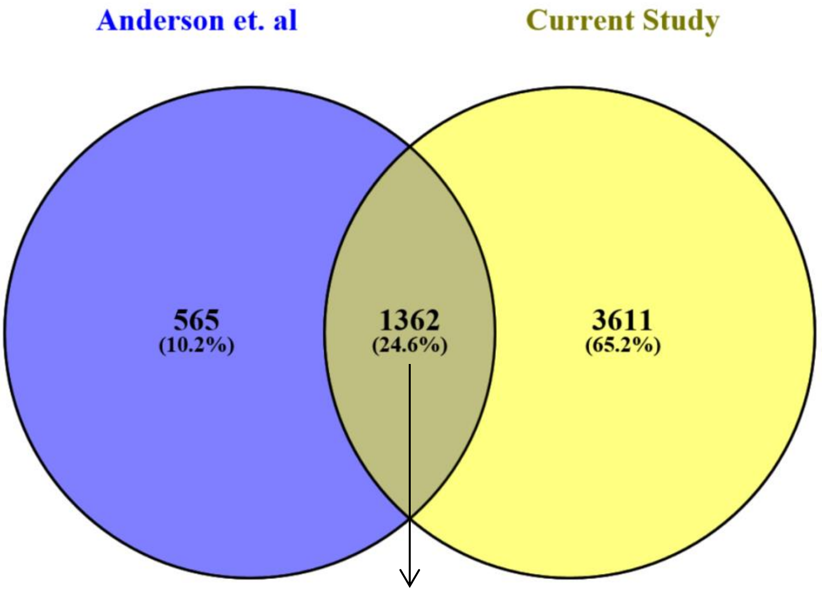

FN1	ITGA3	RPS3A	PKM	DYNC1H1	AHNAK	RAB12	SEC22B	RPS26	ENO2	PSMD2	FRYL	KRT17	GFPT1	IGFBP7	TM9SF2	TMEM104
MFGE8	PDCD6IP	FLNA	UBE2V2	LAMA4	TNC	COL4A1	EVA1A	RHOC	SERPINC1	CPNE3	CRYAB	ATP6V1B2	PSMD6	GDI2	XYLT2	FAM129B
EDIL3	CD63	KRT10	GNB4	CALM3	SLC17A5	ARF4	H2AFY2	TBCEL	NPC1	MXRA7	CORO1C	SQSTM1	SDC2	SLC16A2	KPNB1	EFEMP1
COL1A1	DES	COL12A1	RPL11	GSDMD	RPL36A	FLNB	ACADM	GLIPR2	PSMC5	GOLIM4	PLEKHB2	GANAB	PDIA3	UBL3	YIF1A	MYO1E
COL1A2	NT5E	LAMP2	FLNC	MXRA8	PDGFRB	TSG101	ATP2B4	UGDH	U2AF2	LAMTOR1	YIPF5	SLC7A1	RPL30	RPL9	XRCC5	PLAT
TF	INSL4	LRRC56	RPS23	RAC2	MYH9	RAB35	SLC1A3	COL5A2	GNA13	FASN	SCAMP1	TMEM55A	PLS3	PFKP	EIF4A1	TSPAN2
LGALS3BP	ITGA5	IGSF8	PSMB4	RAC1	MAMDC2	GYG1	ACLY	ALCAM	TTLL12	GPRC5A	DNAJA1	SLC16A1	GPC1	ADK	RUVBL2	MYL12A
ACTB	HIST2H2AC	GJA1	NPTN	RAC3	ENO1	PTRF	NSUN4	RPS19	HNRNPH1	VPS37B	PCBP1	H1F0	EPHB2	FAT1	F2R	MYL9
MVP	HSPA5	YBX1	ITGAV	ITIH4	ATP1B3	PSMB2	SERINC5	LMAN1	CMYA5	PLEC	JAM3	TMED2	RAP1GAP2	CAPZB	TPBG	HRAS
COL6A3	ABI3BP	IGHG1	CD99	RPL13	RAB11B	CCDC102A	ATP2B1	MRC2	MYH14	RFTN1	SNX33	RABEP1	IQGAP1	SYNCRIP	MARCKSL1	ITM2B
COL6A1	EMILIN1	EVA1B	RPL31	RPL26	IGHG2	LDHB	CCDC80	PSMC6	IGFBP3	RPL27	MYO1C	VDAC1	RPL4	FGB	PABPC4	SUMO2
HBA2	MRGPRF	PSMA5	CCT2	CCT8	RAB8A	HLA-B	RPLP2	PSMC3	NTN4	GFPT2	IGF2R	GNG5	CLMP	ACACB	PPIB	AP2A1
ANXA2	TTYH3	FTH1	MUL1	ANXA7	S100A11	RDX	HEG1	IGKC	STX12	ADAMTSL1	PSMD11	CAPNS1	DDAH2	TRIOBP	MB	SND1
RPS27A	PSMA1	RPS3	RAB1A	PSMB7	RPS5	CFB	RPS20	GNAI1	PGAM1	TGFB1	DERA	ALDOC	FAM120A	SRP14	NRAS	CALD1
ACTA2	HPX	ADAM10	LOXL2	PSMA3	TSPAN5	RPL26L1	ATL3	RRAS2	LTBP1	GOLGA3	KRT16	RPL22	ENDOD1	HYOU1	TMEM248	BNIP3
HIST2H4A	RAB7A	SLC1A5	PPIA	S100A6	LAMB1	RPL12	RAB14	TFRC	TSPAN3	BST1	CLIC1	SPTBN1	CNIH4	RECK	PPP1CC	RAB5A
VCAN	ANXA1	TUBB	GNAI2	HSP90AB1	SDC3	RPS17	QRSL1	MATR3	STARD3NL	MPZL1	ITGA2	PPAP2B	PPP1CA	TMEM179B	TCIRG1	CLCN7
A2M	THBS2	TUBB2B	RPS4X	HSP90AA1	TCP1	VPS4A	RHOA	YWHAH	TPI1	AXL	KRT14	ANTXR2	SH3GL1	SNRNP70	PSMB8	NNMT
COL6A2	RTN4	HSPG2	RPL3	CCT6A	TGOLN2	YWHAG	CTNNB1	CANX	ELN	WNT5B	QSOX1	AIMP2	GSTM3	COL14A1	GNA11	EIF3E
TGFBI	MARCKS	RPS6	SLC25A5	MIPEP	RAB30	FAP	SCUBE3	RALA	MFSD11	MYO1B	SEC23IP	ARRDC1	PSMC4	ARPC2	ZYX	UGP2
COL5A1	PTGFRN	RPL7A	ERC1	RPS16	GNAS	GLG1	RPL19	TMED9	PTTG1IP	TMED10	LOX	PI4K2A	DYSF	ATP5B	PSMD8	ATP6V1A
ALB	KRT1	HAPLN3	FMN2	RPL36	RAB8B	YWHAQ	EPRS	EIF3A	COPB2	ATP6AP1	GIPC1	CDH2	RAB23	TPM2	TEX10	SNRPD2
BASP1	SPAG9	RPL15	FBXO28	MYOF	SLC35F6	MYL6	IFITM2	SERPINH1	ACP2	KRT19	CYBRD1	KIF5B	SFT2D2	TPM4	LAMA2	RAB21
HAPLN1	TUBA1C	TUBB4B	KDM5B	SLC7A5	FLOT2	NID2	PSMD1	SPTAN1	WDR1	COPB1	LMBRD1	RAB13	SLC15A4	EGFR	ATIC	SLC20A1
TMOD2	PCOLCE	MCAM	TAGLN	SLC3A2	M6PR	CFI	TMBIM1	EFCAB5	MAPK3	SNRPN	SLC2A3	TLN2	CDKN2AIP	GPX8	SRSF7	SDF4
COL3A1	STOM	POSTN	RAB33B	PSMA2	ARF3	GNA12	RAB3D	ANXA4	LUM	FAM3C	AGRN	CTSD	SLC38A7	TRA2B	EIF5A	CPS1
CD81	HLA-A	RPS2	RAB43	RPL6	BGN	IGLL5	RPL10A	IGHG4	SFN	BROX	ACTR3	TM7SF3	RABAC1	PVR	FABP4	STRAP
HTRA1	NEFM	GNB2	RAB4B	TUBB3	SLC34A3	TMEM106B	IGHG3	TAGLN2	PDCD6	ERGIC1	MYOM3	LMNA	HNRNPA1	OSMR	LDLR	EIF3I
ITGB1	INA	RPL17	RAB4A	RPS4Y2	RPL13A	RPL34	FERMT2	COPA	TMED4	TIMP2	GALNT1	MTR	HNRNPF	SLC2A6	NAPA	SERPINF2
ANXA6	STX7	GNB1	FEN1	CD9	RPL23	RPL10	HNRNPC	YIPF6	DNAJA2	ITGA11	VTI1B	GYS1	SRI	DIRC2	PTPDC1	KRAS
CD59	TUBA4A	ANPEP	PSMA6	ZC3H11A	CRIM1	ARF5	HNRNPCL1	RRAS	ACTR5	AGTRAP	GJC1	PSMD14	GALNT2	JAK2	ATP5G1	CCDC149
ACAN	NRP1	RPS8	HSPB1	APOB	CTNNA1	HAS2	DPYSL3	RPS13	RAB5C	EIF3C	ACTR2	CYFIP1	PHGDH	RPS10	LRRC32	C5orf15
THBS1	KDELR2	KIAA1199	PZP	RPL24	RAB2A	TRAP1	VPS37C	EFEMP2	RPL21	SACS	CD248	ANTXR1	PARD3B	TEP1	PLOD2	SUB1
HSPA2	HIST1H2AB	ADAM9	SLC2A1	MMP2	PRSS23	SYPL1	AIFM1	FHL2	H2AFY	ECE1	SLC7A6	SCAMP2	RAD23B	IARS	COPE	TIMP3
HSPA8	NXF1	EEF1A2	ANXA11	APOE	RAB10	SLC25A6	ABCB6	YWHAB	PLXNB2	TMEM55B	VPS28	PABPC1	ARPC1B	CD109	PAFAH1B1	CHMP6
POTEJ	TMEM256-	RPL35A	RAP1B	SPNS1	EZR	RAB6A	ATP6V0D1	CD47	CD46	MYLK	REEP5	PRDX4	ATP6V1G1	FSCN1	PRAF2	NCL
HIST2H3PS2	PLSCR3	ATP1A1	PLP2	S100A10	MMP14	PSMB5	RAB2B	ACTN1	VAPA	SLIT3	COL4A2	RPL38	ITPR3	PSMC1	FZD7	SORT1
SDCBP	TSPAN4	RPL18	CDC42	CCT5	ALDH16A1	TSPAN14	ANXA8L2	ATOH1	WNT5A	TMEM30A	COPG1	APOC2	ITPR2	SLC39A1	RPS7	RPS21
H3F3B	EEF1A1	CSPG4	ITGB5	HNRNPU	FLOT1	KRT9	CLEC16A	ENPP1	TMEM119	AHCY	ASAH1	RNF167	ITPR1	COPZ1	PPP1CB	CLDND1
HIST1H1B	RPS18	EHD2	NID1	RABL3	PFN1	AUTS2	GNAI3	SMC2	EPHA5	NPM1	CD320	VAMP5	ACTR1B	PLS1	NME1	PVRL2
HIST1H2BL	HSPA9	PXDN	ALDOA	DHX8	CFL2	EPHA2	PRDX1	IGLC2	MYH10	F5	SLC31A1	SLC29A1	CHMP2A	ITGA6	NME1-	DCN
HIST1H2BO	FBN1	EHD3	HBB	RAP1A	ENG	RPS15A	PRPF38B	NQO1	LTBP2	NCKAP1	IGHA1	ARL8A	DPYSL2	DDR2	NME2	VAPB
HSPA1A	BSG	KRT2	CLIC4	TSPAN6	CAD	RP11-	PDGFRA	THBS3	CTSB	RALY	P4HB	SERPINE1	ARHGDIA	RPL32	NDFIP2	PDLIM5
VIM	PRPH	KRT6B	HBD	CCT4	C12orf75	1035H13.3	ACTR1A	SLC43A3	HSPD1	ADC	XRCC6	GOLGA7	RP11-	STXBP1	RPL37	SF3A1
GAPDH	SCARB2	RPL5	RPL8	KRT6C	RHOG	NOTCH2	OLFML3	CLTA	TMF1	PCBP2	AZGP1	RPS29	762I7.5	RRBP1	PSMD12	THRAP3
UBR2	KRT7	FTL	GSN	RPL18A	PODXL	SLC44A1	ARG1	RAN	RPSA	PGK1	CDH13	CACNA2D1	MTMR6	TMEM200A	PPP2R1A	DHX9
HIST1H1C	KRT8	PSMB6	VAMP3	LGALS1	RPS9	VASN	PTK7	MFSD1	TMEM165	SNRPF	GSTM4	TOM1	PLOD1	SNAP23	AP2B1	ILK
HIST1H1E	CLTC	SERPINE2	PSMA4	RAB1B	PSMC2	TLN1	ARL8B	C4A	PSMD3	CNP	GNB2L1	FHL1	MUC4	LAMTOR5	DCBLD2	RAB5B
CD44	SLC38A2	RPS14	TYRP1	CCT7	RPLP0	SERINC1	KRT77	VCL	HNRNPK	CALR	SERPINA1	APLP2	BMP1	CAPZA1	VAMP7	EDF1
ANXA5	FBLN1	PSMB1	RPL7	CD151	PLAUR	PSMD13	PRNP	DSTN	IST1	RPS27L	RPL37A	PRKDC	TMEM2	ATP6V0A1	ARL6IP5	PRDX3
KIAA1217	LRP1	RPL28	VAMP2	NUP54	GNG12	C3	ITIH3	ITGB3	HNRNPUL2	RPS27	CTNND1	LAMTOR2	SEMA7A	CPNE1	NOTCH3	ADAM12
LAMP1	RPL29	KRT84	CCT3	SCAMP3	KRT5	SLC16A3	ARF6	LMAN2	-BSCL2	APOA1	EHD4	STC2	TUBB6	PUF60	CSRP1	TMEM9
SSC5D	PARP4	VCP	EEF2	SLC44A2	RPL35	RPL18	RAB3B	CD276	COL8A1	TSPAN9	BCAP31	TMED7-	HGS	CD99L2	RPS24	SREBF2
TPP2	EHD1	MSN	LDHA	PSMB3	RPS25	TKT	SRSF3	UBR4	JTB	SRSF1	TGM2	TICAM2	CD82	PTBP1	LNPEP	SLC8A1
MINK1	H2AFX	CAV1	SLC1A4	CFL1	PSMA7	EEF1G	SLC6A6	RPL27A	STX4	CACNA1I	ACTN4	CAP1	TMEM181	ACTR3B	HNRNPR	ATP6V1E1
HNRNPL	LAMC1	RPS11	PTX3	YWHAZ	RPL36AL	MYL6B	RPL14	YWHAE	HNRNPH2	TM9SF3	HSP90B1	INTS1	CYB5R3	SLC39A14	LPAR1	G6PD
DARS	TVP23C	MFAP5	TNS1	PTPRS	LIN7C	UEVLD	MACF1	TMEM109	HADHB	CEP350	FXR1	TMEM87B	VPS29	HNRNPA2B1	ITFG3	CD97
PEF1	MFSD8	SLC12A4	PALLD	PEAR1	EIF3D	RELL1	C1S	ILF2	CLU	VPS37A	PITPNB	HMCN1	ACOT9	GLUD1	CHMP5	RTN3
FMNL2	ABI1	SLC26A11	DDX39A	CALU	ARPC1A	SEH1L	PRPF8	FSTL1	ADAMTS1	STAM2	STAT1	VPS4B	SLC39A6	NONO	HGSNAT	STEAP3
SELENBP1	FZD6	SF1	GABARAP	TINAGL1	SH3BP4	PRKAR2A	VASP	BTN2A1	LIMS1	GAS6	DAB2	MLF2	PLXNA1	RAP2C	UBA1	MAPK1
FARP1	SCUBE2	DYNC1I2	STXBP3	EFNB1	PLIN3	ITCH	RHEB	CC2D1A	MTA2	CTSZ	NUTF2	SMURF2	ALG5	ATP2A2	RARS	PRDX2
TPM3	FAS	LRRIQ1	JUP	ARRDC3	SGMS2	PTP4A2	ZDHHC20	CAPZA2	LAMTOR3	FAM26E	NOTCH1	LRP12	ADSL	JAK1	RAP2B	PLVAP
LAPTM4A	ORM1	DNPEP	TMEM59	TOP1	TMX1	PIEZO1	MAN2A1	TMEM173	ASPN	PTPRG	SLC12A6	TNS3	UBE2N	YIPF4	DIP2B	BAG2
KIAA1715	ATP1B1	CAMK2D	IGFBP4	RALB	PCDHGA12	SLC2A13	HIATL1	BET1L	QARS	COPS5	NIPBL	SUPT6H	DNAJB4	RUVBL1	LPP	MARS
KIRREL	NPDC1	DAG1	CLTB	PCDHGB4	PCDHGC3	CCP110	PELP1	ADH5	MPG	TARS	HK1	INHBA	YKT6	NRBP1	GALK1	SGCD
SERPINB1	SSBP1	RAB32	HPS3	THY1	UBXN1	NEO1	LASP1	IMPDH1	PSME1	GPI	SLC20A2	OLFML2B	KCMF1	VPS13D	IMPAD1	EFNB2
ITM2C	ERBB2IP	NUCB2	SARS	FST	GTF2E2	EIF3F	PAPPA	SRRM1	SLC12A9	PTPN23	SHISA2	PRPS1	ATP2C1	FGA	AP4E1	ERP44
F13A1	U2AF1	UCHL1	NDRG1	SLC2A10	GPC4	PGM1	GOLM1	SRSF6	EIF5B	TXN	GALNT10	OLA1	DCUN1D3	TGFBR2	PRKAR1A	WLS
AP2M1	AKR1CL1	STUB1	CAPN2	C7orf55	ECHS1	TMED3	PLAU	HP1BP3	ATP6V1H	EIF2S3	SLC12A2	UBA6	SF3B3	FMNL3	GSPT1	GPR176
DDX5	DSP	SLC4A4	RPL23A	LUC7L	APOH	NPR3	ATP11C	FERMT1	SAFB	CTSA	LARS	VAMP4	PGD	EPHB4	FGG	CTPS2
ARPC4	SLC9A3R2	DYNC1LI1	STAM	SEC13	ANGPTL4	VAT1	RABGGTA	TSPAN10	SAFB2	FBLN2	WDR26	GEMIN4	KHDRBS1	COL25A1	RPS12	RHBDF2
TENM3	MGAT2	IQGAP3	HINT1	PCDHGA11	ARPC5	FYN	ACSL4	STX8	ATP6V1C1	DLAT	TGFBR1	TXNDC5	ROCK2	PYGB	ACO1	VPS25
IL6ST	ZDHHC5	MFAP2	CYR61	PFKL	CMSS1	LRP10	BCAP29	TRIM28	CYSTM1	WASF2	LMNB1	ADAM17	PAICS	MVB12A	TMEM87A	PGRMC1
GREM1	B2M	RAB9A	RAPGEF3	KRT18	IPO5	DOCK7	TUSC3	JAG1	PDCD1LG2	AP1B1	PICALM	HDAC1	EPHX1	ADH1B	IER3IP1	DLST
MT2A	CKAP4	BMPR2	ARID3A	S100A16	ANKH	PACSIN3	GC	MVB12B	PAH	SMC1A	SERINC3	YBX3	PIP	ACVR1	TMEM106A	RAB31
PRPF40A	DAB2IP	CTPS1	AIDA	CNN2	PLSCR1	ITGA10	ARFIP1	EXT2	PLXND1	GARS	BCLAF1	YIF1B	ESYT1	PLEK	GSTP1	ILF3
EIF3L	RHOB	H2AFV	WDFY1	GPR107	S100A13	SPTLC1	GRPEL1	CLCN3	BST2	VTI1A	PFKM	CTGF	ENAH	ATP5A1	EIF3B	HDAC2
ABI2	SFPQ	SLC39A10	GSTK1	PGK2	ARCN1	TMEM230	SLC4A2	PRPF19	C1GALT1	MAP1LC3B	COMP	CPD	STARD3	TPM1	SH3BGRL	HP
PSMD7	PAPSS2	TSPAN13	AEBP1	SEC31A	SIRPA	SNX17	SLCO3A1	NTM	SLC38A9	OSTM1	CHMP4B	NCSTN	DACT1	TENC1	ITGA7	MAP4K4
ALDH8A1	PPL	ITGA4	IGHV3OR1	VANGL1	ATP8B1	CRIP2	TM9SF4	SYT11	DDX39B	ATRN	PAPSS1	GNAQ	VPS35	TLDC1	RNH1	MCOLN1
		DDX17						DCTN2	PTBP3	TM9SF1	NUCB1	HTRA3	SLK		DCTN1	
